# Cardiac-specific biomarkers and life-threatening complications of off-pump versus on-pump coronary bypass surgery in Egyptian patients

**DOI:** 10.1186/cc11076

**Published:** 2012-03-20

**Authors:** H Elabd, A Alsherif, T El Gohary, M Hagras, S Salah Eldin

**Affiliations:** 1 Student Hospital, Cairo University, Giza, Egypt; 2 Kasr Alaini Hospitals, Cairo, Egypt

## Introduction

Coronary artery bypass grafting (CABG) has traditionally been performed with the use of cardiopulmonary bypass (ONCAB). This study aims to compare between on-pump and off-pump surgery concerning postoperative morbidity and mortality, and also to evaluate 6-month graft patency in Egyptian patients.

## Methods

This nonrandomized single-centre control trial was prospectively conducted on 65 patients who were subjected to coronary artery bypass surgery followed by stay in the Open Heart Intensive Care Center of the Police Authority Hospital, in the period from July 2009 to January 2010. Patients were divided into two groups; group A, 25 patients underwent surgery using cardiopulmonary bypass pump (on coronary artery bypass pump (ONCAB)); and group B, 40 patients underwent surgery without using cardiopulmonary bypass pump (off-pump coronary artery bypass (OPCAB)). All of the demographic, operative and postoperative data were prospectively collected and analyzed statistically. Six months later, the patients underwent coronary angiography.

## Results

There was no significant difference between both groups intraoperatively concerning arrhythmias, blood transfusion, and hemodynamic support. Off-pump patients had a significantly higher mean number of constructed grafts than in the ONCAB group (mean, 3.30 ± 0.88 vs. 2.84 ± 0.80, *P *= 0.02). There were no significant differences between off-pump and on-pump regarding postoperative blood loss, blood transfusion, length of the ICU and the hospital stay, the ventilation time, the use of intraaortic balloon pump, renal complications, respiratory complications, and reopening. However, graft occlusion, MI, raised cardiac enzymes, ventricular tachycardia, cardiogenic shock, and disturbed conscious level were significantly higher in the OPCAB group. The postoperative mortality rate was significantly higher in the OPCAB group than in the ONCAB group (15% vs. 0%, *P *= 0.046). Follow-up angiograms in 40 patients out of 65 (61.5%) who underwent 124 grafts revealed that no significant difference between off-pump and on-pump groups regarding the overall rate of graft patency (83.5% vs. 84.4%, *P *= 0.84). No mortality was reported in both groups at 6-month follow-up.

## Conclusion

There was a higher incidence in postoperative complications and mortality in the off-pump procedure than the on-pump. At 6-month follow-up, no significant differences between both techniques were found in graft patency and mortality. Hence, longer-term mortality from randomized trials of off-pump versus on-pump CABG is needed.
